# PopTradeOff: A database for exploring population-specificity of adaptive evolution, disease susceptibility, and drug responsiveness

**DOI:** 10.1016/j.csbj.2023.06.008

**Published:** 2023-06-10

**Authors:** Ji Tang, Huanlin Zhang, Hai Zhang, Hao Zhu

**Affiliations:** a Bioinformatics Section, School of Basic Medical Sciences, Southern Medical University, Guangzhou 510515, China; b Network Center, Southern Medical University, Guangzhou 510515, China; c Guangdong-Hong Kong-Macao Greater Bay Area Center for Brain Science and Brain-Inspired Intelligence, Southern Medical University, Guangzhou 510515, China; d Guangdong Provincial Key Lab of Single Cell Technology and Application, Southern Medical University, Guangzhou 510515, China

**Keywords:** Database, Favored mutation, GWAS, Disease susceptibility, Drug responsiveness

## Abstract

The influence of adaptive evolution on disease susceptibility has drawn attention; however, the extent of the influence, whether favored mutations also influence drug responses, and whether the associations between the three are population-specific remain unknown. Using a reported deep learning network to integrate seven statistical tests for detecting selection signals, we predicted favored mutations in the genomes of 17 human populations and integrated these favored mutations with reported GWAS sites and drug response-related variants into the database PopTradeOff (http://www.gaemons.net/PopFMIntro). The database also contains genome annotation information on the SNP, sequence, gene, and pathway levels. The preliminary data analyses suggest that substantial associations exist between adaptive evolution, disease susceptibility, and drug responses and that the associations are highly population-specific. The database may be valuable for disease studies, drug development, and personalized medicine.

## 1 Introduction

Abundant data generated by genome-wide association studies (GWAS) and clinical drug research have revealed the population specificity of many GWAS sites and drug response-related sites [3], [4], [1], [2]. How the population specificity is determined and whether disease susceptibility and drug responsiveness have intrinsic associations are important questions for basic, translational, and clinical biomedical research. A sensible conjecture is that both are related to the adaptive evolution of humans. This conjecture is supported by findings such as genetic ancestry plays a central role in population pharmacogenomics [5]. However, few systematic investigations have been reported.

**Fig. 1:**
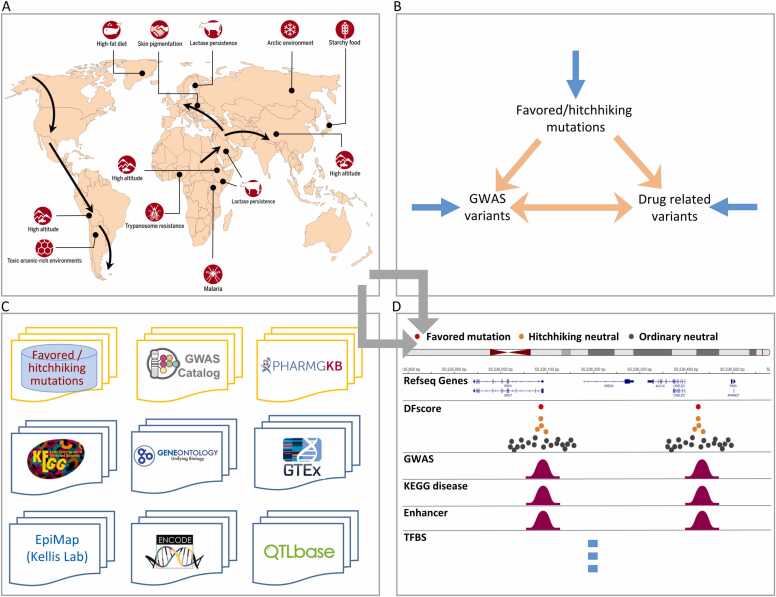
An overview of PopTradeOff. (A) Many population-specific mutations occurred during and after the out-of-Africa migration, making humans adapt to varied local environments and changed lifestyles but also susceptible to particular diseases (note that the 17 populations covering Asians, Europeans, and Africans are not really worldwide; thus, some favored mutations in this panel are not in the database) (adapted from [9], [7]). (B) PopTradeOff helps examine the associations between the three families of mutations. Blue arrows indicate the traditional studies, including population genetics, GWAS study, and pharmacogenetics. Orange arrows indicate the combined studies. (C) PopTradeOff contains data from multiple sources. (D) An illustration of the genome browser webpage that displays a search result in multiple tracks.

During and after the migration out of Africa, many single nucleotide mutations occurred in the genomes of different populations, allowing these populations to adapt to different environments and lifestyles (Fig. 1A) [6], [7], [8]. These mutations are called beneficial (favored) and influence phenotypic differences between human populations. However, recently, evidence has emerged suggesting that many favored mutations have been selected with a cost; that is, they also make the carriers susceptible to certain diseases [10], [11], [9]. Increasing findings indicate that favored mutations are both population- and disease-associated. For example, the mutations in the *HBB* gene make some Africans resistant to *P. falciparum* malaria but also susceptible to sickle cell anemia [12], and the mutations in genes encoding hypoxia-inducible factors (HIFs) make the carriers benefit from increased oxygen delivery but also suffer from increased blood viscosity (a contributing factor to the high incidence of stroke in Tibetans [13]. These findings provide the primary support for the conjecture that population-specific favored mutations critically associate the relationships between adaptive evolution, disease susceptibility, and drug responses (Fig. 1B). Deciphering the association helps researchers understand human evolution and physiology and improves personalized medicine.

Identifying favored mutations has been a challenge for decades. Many statistical tests have been developed to detect signals in the genome generated by favored mutations (called selection signals, which are short genomic regions in varied lengths and with varied features) [14]. However, detecting the causal mutations (i.e., favored mutations) is much more difficult, and integrating multiple statistical tests is a highly recognized strategy [15], [16]. Recently, we used a deep learning network (called DeepFavored) to integrate seven statistical tests to systematically identify favored mutations in the human population CEU, CHB, and YRI [11]. DeepFavored outperforms other integration methods, and the combined analysis of favored mutations and GWAS sites reveals the intrinsic association (i.e., extensive trade-offs) between population-specific adaptive evolution and disease susceptibility [11].

In this study, we further trained the DeepFavored network using simulated population genomic data of 17 European, Asian, and African populations and used the trained network to identify favored mutations in the 17 populations. Further, we integrated favored and hitchhiking neutral mutations (hereafter hitchhiking neutral mutations and ordinary neutral mutations are simply called hitchhiking mutations and ordinary mutations, respectively) with disease/trait phenotype and drug response phenotype data into the database PopTradeOff (Fig. 1 C). This database helps unveil the associations between adaptive evolution, disease susceptibility, and drug responsiveness to study human physiology and disease and perform precision and personalized medicine. The preliminary data analysis indicates that many favored and hitchhiking mutations influence gene expression and function, disease susceptibility, and drug responses population specifically (Fig. 1D).

## 2 Materials and methods

### 2.1 Identifying favored mutations in 17 populations

First, we downloaded the genome-wide mutation data (excluding the Y chromosome) of 17 European, Asian, and African populations from the 1000 Genomes Project (Phase 3, GRCh37/hg19) [6]. The European populations include Utah residents with Northern and Western European ancestry (CEU), Finnish in Finland (FIN), British in England and Scotland (GBR), Iberian populations in Spain (IBS), and Toscani in Italia (TSI). The Asian populations include Han Chinese in Beijing (CHB), Chinese Dai in Xishuangbanna (CDX), Southern Han Chinese (CHS), Japanese in Tokyo (JPT), and Kinh Vietnamese (KHV). The African populations include Yoruba in Ibadan, Nigeria (YRI), African Caribbean in Barbados (ACB), Luhya in Webuye, Kenya (LWK), Mende in Sierra Leone (MSL), African Ancestry in Southwest US (ASW), Esan in Nigeria (ESN), Gambian in Western Division, and The Gambia (GWD). The ID lists of unrelated individuals were acquired from the Ensembl website [17].

Second, we used the demographic models of European, Asian, and African to simulate population genomic data [18], [19], used the simulated population genomic data to train the deep learning network DeepFavored, and used the trained network to identify favored and hitchhiking mutations in the 17 populations. As reported [11], the trained network integrates the seven statistical tests Fst, XPEHH, iHS, ΔDAF, nSL, iSAFE, and ΔiHH.

Third, favored and hitchhiking mutations in each population were predicted upon scores that reflect the strength of positive selection (called DFscore), together with the ranks of the scores in 600 KB genomic regions centered on these mutations. Previous studies suggest that 600 KB is the size of genomic regions affected by a moderately strong selection [16]. Mutations with DFscore> = 0.5 and rank< = 3 (in each 600 KB window) were identified as favored mutations, and nearby neutral mutations (within the 600 KB window) with linkage disequilibrium (LD)> 0.6 with the favored mutation were identified as hitchhiking mutations. Analysis using simulated data indicates that the probability that a neutral mutation gets a DFscore> 0.5 is< 1e-6. Finally, correlation coefficients were calculated to measure the likelihood that favored alleles and the alleles in hitchhiking sites are on the same haplotypes.

### 2.2 Integrating favored/hitchhiking mutations with trait/disease and drug response data

We downloaded favored/hitchhiking mutation-related disease/trait and drug response data from the GWAS Catalog (https://www.ebi.ac.uk/gwas/) [2] (version 1.0.2, including about 150000 GWAS sites with p value<1e-5), PharmGKB (https://www.pharmgkb.org/) [3], [20], and DrugBank (https://go.drugbank.com/) databases [4]. The GWAS Central database also contains many GWAS sites and can be used as an external data source (https://www.gwascentral.org/) [1].

### 2.3 Collecting genome annotation data

Genome annotation data include the Ensembl genome annotation (GRCh37 release 87) downloaded from the Ensembl website [17], the pathway information downloaded from the KEGG website [21], and the gene set information downloaded from the Gene Ontology (GO) website [23], [22]. Disease annotation data were downloaded from the KEGG Disease database [21]. Quantitative trait loci data across multiple human molecular phenotypes were downloaded from the QTLbase website [24]. The annotation of enhancers, including 2.1 million active enhancers and 3.3 million tissue-specific enhancer-gene links, was downloaded from the EpiMap website [25]. The transcription factor (TF) binding sites (TFBS) data, which contain 338 TFs and 130 cell types generated by the ENCODE project (1264 ChIP-seq experiments) [26], were downloaded from the UCSC Genome Browser (http://genome.ucsc.edu/). For each TFBS, the nearest genes were treated as the target genes of the TF, and the 130 cell types were classified into 33 groups upon the *main_metadata_table.tsv* in the EpiMap website. The four families of data (favored/hitchhiking mutations, GWAS sites, drug response-related sites, genome annotation data) were integrated into PopTradeOff database using the MySQL software.

### 2.4 Developing the data search and display functions

The user interface of PopTradeOff was built using the Django (https://docs.djangoproject.com/en/4.1/) and VUE frameworks (https://vuejs.org/guide/introduction.html). Data in PopTradeOff can be searched for upon flexibly combined conditions. The igv.js software was revised and used to develop the genome browser that graphically displays search results in UCSC Genome Browser-like tracts (Fig. 1D) [27].

## 3 Results

### 3.1 Overview of data

The trained deep learning network DeepFavored outputs a score (called DFscore) for each mutation in the genome of each population and a rank of the score in a 600 KB window centered on the mutation [11]. DFscore> = 0.5 and rank< = 3 strongly suggest favored mutations. Upon the stringent criteria DFscore> = 0.5 and rank< = 3, 21293 favored mutations were identified in the 17 populations, and the probability a neutral mutation meets the criteria is< 1e-6. Then, in windows containing favored mutations, 867379 neutral mutations having linkage disequilibrium (LD)> 0.6 with favored mutations were identified as hitchhiking mutations.

**Fig. 2:**
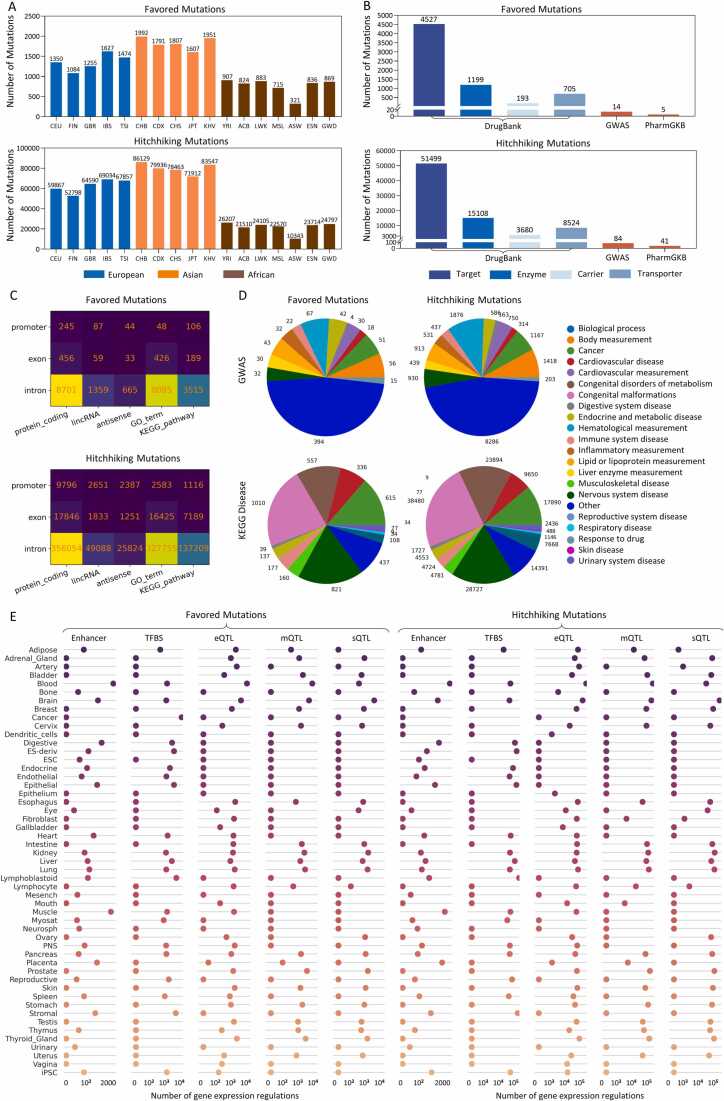
Data summary of PopTradeOff. (A) The number of favored and hitchhiking mutations in the 17 European, Asian, and African populations. (B) The number of favored and hitchhiking mutations that overlap drug response-related sites. The ‘GWAS’ bars indicate only the GWAS sites associated with drug responses. Drug response-related genes in DrugBank were categorized into four classes: Target, Enzyme, Carrier, and Transporter. (C) The number of favored and hitchhiking mutations located in different genomic regions, gene types, gene sets, and pathways. (D) The number of favored and hitchhiking mutations potentially related to different diseases. (E) Favored and hitchhiking mutations at different regulatory loci influence tissue-specific gene expression. The horizontal axes denote the numbers of target genes of these regulatory loci in specific tissues.

**Fig. 3:**
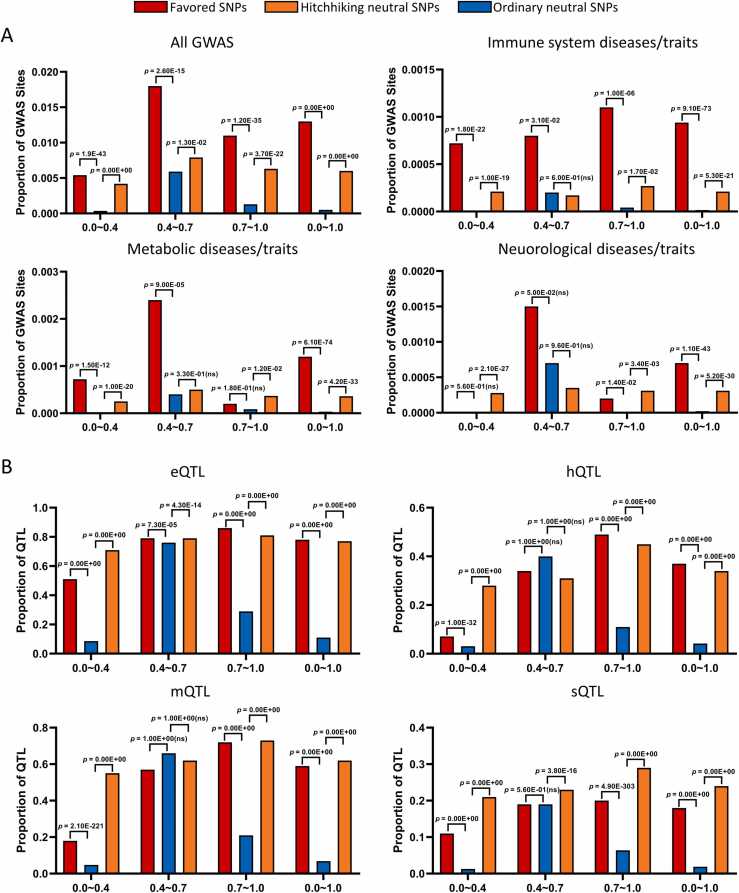
Enrichment of favored and hitchhiking mutations in GWAS sites and QTLs. Red, orange, and blue bars represent favored, hitchhiking, and ordinary mutations. The statistical significance of enrichment across different derived allele frequency bins (numbers in the horizontal axis) is examined by one-side two-proportion Z test (‘ns’ indicates insignificance). (A) Favored and hitchhiking mutations are especially enriched in GWAS sites associated with nervous, immune, and metabolic system diseases or traits. (B) Favored and hitchhiking mutations are enriched in multiple types of QTLs, including expression quantitative trait loci (eQTL), methylation quantitative trait loci (mQTL), histone quantitative trait loci (hQTL), and splicing quantitative trait loci (sQTL).

Favored and hitchhiking mutations in the 17 populations have several features (Fig. 2, Fig. 3). First, more favored and hitchhiking mutations are identified in Asian and European populations than in African populations, and most favored and hitchhiking mutations are population-specific. The two findings are reasonable because Asian and European populations have experienced more recent adaptive evolution events than African populations. Second, many favored and hitchhiking mutations overlap GWAS sites in the GWAS Catalog database and drug response-related sites in the DrugBank and PharmGKB databases. There are 5353 overlaps between favored mutations and GWAS sites/KEGG disease genes (and 178568 overlaps between hitchhiking mutations and GWAS sites/KEGG disease genes), and the overlaps with GWAS sites show statistical significance. Third, favored and hitchhiking mutations are especially enriched in GWAS sites associated with nervous, immune, and metabolic systems, agreeing that these systems have experienced significant evolution (Fig. 3A). Fourth, many favored and hitchhiking mutations also overlap multiple types of regulatory sites with statistical significance (Fig. 3B). Fifth, the tissue distribution of the target genes of these regulatory sites indicates that favored and hitchhiking mutations influence gene expression tissue specifically (Fig. 2E). Sixth, favored and hitchhiking mutations that overlap drug response-related sites are limited. Nevertheless, many favored and hitchhiking mutations are in exons of and quantitative trait loci (QTLs) in drug response-related genes. The above features suggest that adaptive evolution considerably influences gene expression and disease susceptibility and that adaptive evolution may influence drug responses on the gene level.

### 3.2 Key functions

#### 3.2.1 Search engine

A search engine is developed for searching favored mutations, hitchhiking mutations, GWAS sites, and drug response-related sites. By searching data upon flexibly defined conditions, the database can be used to identify (i) favored and hitchhiking mutations and genes influenced by adaptive evolution in the 17 human populations, (ii) whether these genes influence drug responses and actions in a population-specific manner, (iii) whether these genes influence the susceptibility/severity of specific diseases, (iv) the distribution of disease- and drug-related favored/hitchhiking mutations in specific genomic regions. The results of a search are displayed in a Result table.

#### 3.2.2 Genome browser

**Fig. 4:**
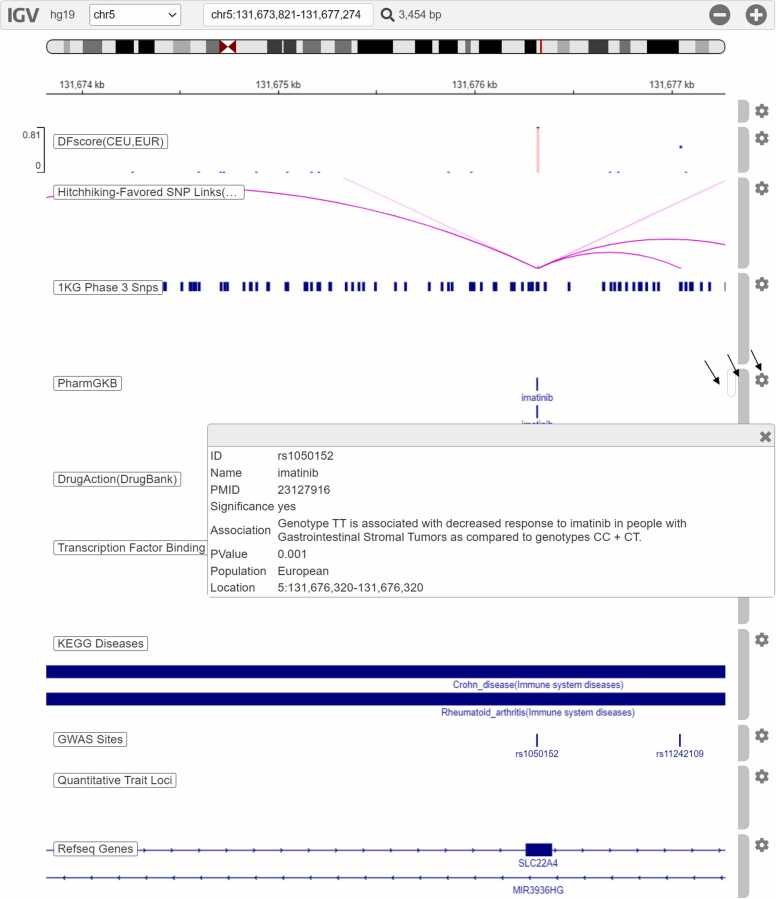
The genome browser webpage shows some tracks of rs1050152. Here rs1050152 is a search result under the combined conditions (i) "Specify a mutation type" = " Favored", (ii) "Specify a population" = "All", (iii) "Input a KEGG disease ID or a keyword" = " crohn". The left side displays track names. On the right side are track-specific vertical scroll bars (each track has its own scroll bar for displaying sub-tracks), track highlighting bars, and track configuration buttons (indicated by arrows). The "DFscore" track shows that the mutation has a high DFscore in the population CEU and super-population EUR. The "Hitchhiking-Favored SNP Links" track shows hitchhiking mutations of this mutation. The "1KG Phase 3 Snp" track shows the SNPs of 1000 Human Genome. Clicking one of "imatinib" records in the PharmGKB track, a window pops up and displays the content of the PharmGKB record. The "KEGG Diseases" track shows that this mutation is involved in Crohn's disease and Rheumatoid arthritis. The "GWAS Sites" track indicates that this mutation is a GWAS site. This example demonstrates the associations between adaptive evolution, disease susceptibility, and drug responsiveness.

To look into details of a record in the Result table, click that record to open the genome browser. The graphical genome browser displays each mutation and relevant attributes in tracks in the context of the human genome hg19. The genome background shows the information that a mutation plays its role. The order, height, and color of tracks can be configured flexibly. Clicking the stripes, points, or arcs in the tracks can open popup windows to show more details (Fig. 4).

#### 3.2.3 Drug response information

Some mutations have many drug response-related records in PharmGKB and DrugBank. To display these records' details, click the record names in the PharmGKB and DrugBank tracks to open popup windows.

### 3.3 Examples of associations between adaptive evolution, disease susceptibility, and drug responses

Here we present several examples to illustrate using PopTradeOff to explore the associations between adaptive evolution, disease susceptibility, and drug responses.

#### 3.3.1 The immune disease-related rs1050152 hosts a favored mutation in European populations

rs1050152 is an immune disease-related variant in some European populations. The related diseases include Crohn's disease, asthma, and ulcerative colitis (https://www.gwascentral.org/marker/HGVM236012/results). As shown in Fig. 4, it is also a drug response-related variant, as genotype CT is associated with an increased response to Ustekinumab in people with Psoriasis as compared to genotype CC (p-value = 0.037), and genotype TT is associated with a decreased response to Imatinib in people (especially Europeans) with gastrointestinal stromal tumors, leukemia, and myelogenous as compared to genotypes CC + CT (https://www.pharmgkb.org/variant/PA166156750/variantAnnotation). Ustekinumab is used to treat immune-related diseases (e.g., plaque psoriasis and Crohn's disease), and Imatinib is used to treat certain types of leukemia, skin cancer, and gastrointestinal stromal tumors. The deep learning network DeepFavored identifies the mutation T on rs1050152 as a strongly favored mutation in CEU (DFscore = 0.815). The predicted favored mutation T, together with the GWAS and PharmGKB data, highlight that this mutation may have been selected for with changed susceptibility to these immune-related diseases and altered responsiveness to the related drugs.

#### 3.3.2 The scalp hair shape-related rs11150606 hosts a favored mutation in East Asians

rs11150606 is a phenotype-related variant in East Asian populations. The related phenotype is scalp hair shape (https://www.gwascentral.org/marker/HGVM3858600/results). It is also a drug response-related variant, as allele C is associated with a decreased dose of Warfarin as compared to allele T (p = 0.0221) (https://www.pharmgkb.org/variant/PA166155104/variantAnnotation). Warfarin is used to prevent blood clots from forming or growing larger in blood and blood vessels. The mutation C on rs11150606 has a high frequency (>76.4 %) in East Asian populations but a low frequency (<20.1 %) in other populations. The deep learning network DeepFavored identifies the mutation C on rs11150606 as a strongly favored mutation in East Asians (DFscore = 0.713/0.795/0.651/0.667 in CDX/CHS/JPT/KHV, respectively). The predicted favorable mutations together with the GWAS and PharmGKB data highlight that positive selection for the favorable mutation C may be associated with scalp hair shape and warfarin responsiveness.

#### 3.3.3 rs776746 contributes greatly to inter-population variation in drug metabolism

rs776746 is an SNP encoding the (nonfunctional) CYP3A5 * 3 allele of the *CYP3A5* gene. Because the *CYP3A5* gene encodes a member of the cytochrome P450 superfamily of enzymes, the cytochrome P450 proteins catalyze the metabolism of many drugs and the synthesis of cholesterol, steroids, and other lipids, CYP3A5 is an important contributor to inter-population variation in drug metabolism. Of note, the frequency of the C allele is low in AFR but high in all other populations (especially in Europeans), and the frequency of the T allele shows the opposite (high in AFR but low in all other populations, especially in Europeans). While the related GWAS phenotypes are limited, including "glycated hemoglobin levels" and "Tacrolimus trough concentration in kidney transplant patients" (https://www.gwascentral.org/marker/HGVM696974/results), many studies reported rs776746 as a drug response-related variant (https://www.pharmgkb.org/variant/PA166157267/variantAnnotation). Here are some important cases. (i) Genotype CC is associated with a reduced dose (or increased risk) of tacrolimus compared with genotypes CT+TT in kidney transplant patients (p < 0.001), and genotype TT is associated with an increased dose of Tacrolimus compared with genotype CC in kidney transplantation patients (p < 0.0001) [28], [29], [30]. (ii) Genotype CC is associated with increased metabolism of amlodipine compared with genotypes CT + TT in healthy individuals [31]. (iii) Genotypes CC + CT are associated with increased response to Atorvastatin compared with genotype TT [32], [33], [34]. (iv) Genotype CC is associated with a decreased dose of cyclosporine compared with genotypes CT + TT [35]. (v) Genotype TT is associated with an increased risk of kidney diseases compared with genotypes CC + CT when treated with Dihydropyridine derivatives [36]. The deep learning network DeepFavored identifies the mutation T on rs776746 as a favored mutation in the European population IBS.

## 4 Discussion

Multiple databases, including dbPHSP [37], 1000 Genomes Selection Browser 1.0 [38], and PopHumanScan [39], have been developed for studying selection signals in human populations. However, the favored mutations that cause these selection signals matter more for precision and personalized medicine, and most favored mutations in human populations remain unknown. Here we report the identification of favored mutations in 17 human populations, the development of a database that contains predicted favored mutations, and the integration of the database with GWAS data, drug response-related data, and genome annotation data. PopTradeOff is different from other databases in that it contains favored mutations in multiple populations and integrates favored mutations with three other kinds of data, making it help reveal associations between adaptive evolution, disease susceptibility, and drug responsiveness and serve the studies of diseases.

**Fig. 5:**
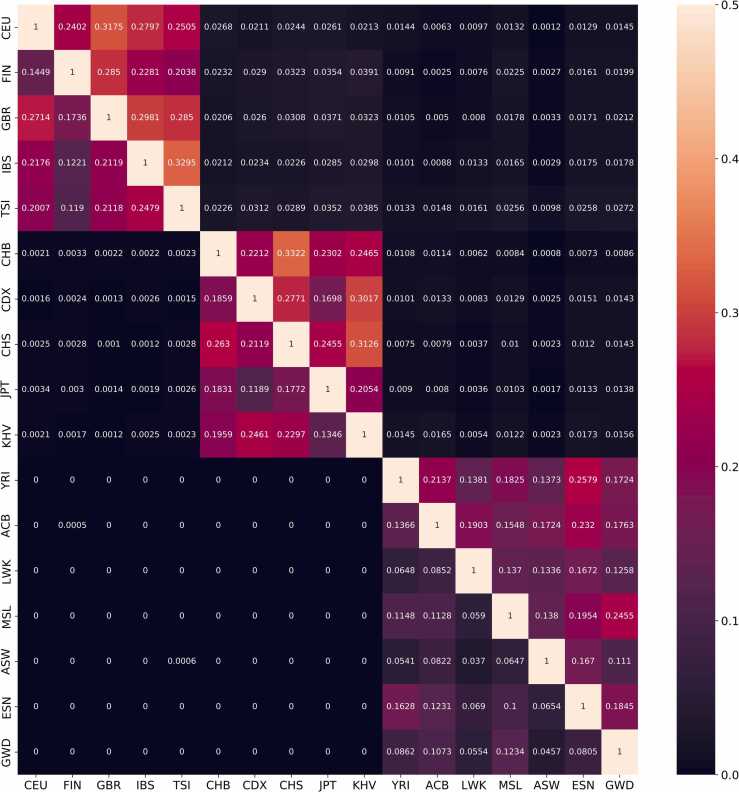
The extent favored and hitchhiking mutations are shared between different populations. The number in each unit denotes the proportion of favored mutations (the left-bottom part) and hitchhiking mutations (the upper-right part) shared between two populations.

To test the population specificity of the association, we examined the extent favored and hitchhiking mutations are shared between populations. We found that a higher proportion of favored and hitchhiking mutations is shared between populations of the same group (Asians, Europeans, or Africans) and that fewer favored mutations are identified in African populations (Fig. 5). These findings are reasonable for two reasons and somewhat validate the favored mutation prediction. First, populations of the same group (e.g., Asians or Europeans) have the same or close ancestries. Second, there are more young long-range haplotypes in populations that emigrated from Africa than in African populations. Because recombination events progressively disrupt long-range haplotypes, young long-range haplotypes are more likely to remain selection signals than old ones, contributing to more favored mutations being detected in Asian and European populations.

The association between adaptive evolution and disease susceptibility has drawn researchers' attention [10], [9]. Our previous analyses of favored and hitchhiking mutations and GWAS sites suggest that there is an extensive and population-specific trade-off between adaptive evolution and disease susceptibility and that disease-related favored mutations especially influence metabolism, immune, and nervous system diseases (these systems have evolved substantially during human evolution) [11]. Preliminary analyses of data in PopTradeOff suggest that the association extends to population-specific drug responsiveness. These findings point to the necessity of identifying favored mutations in more populations and integrating data from multiple sources. Exploring and understanding the associations between the three aspects may greatly promote disease studies, drug development, and personalized medicine.

Several notes on the current version of PopTradeOff. First, although we have identified substantial overlap between favored/hitchhiking mutations and GWAS sites, the overlaps between favored/hitchhiking mutations and drug response-related variants reported in PharmGKB are limited. Probably more drug response-related variants are to be identified. Second, many favored/hitchhiking mutations are found in exons of and QTLs in drug response-related genes, suggesting associations at the gene level. Third, the favored mutations in the 17 populations may likely be underestimated because we were prudential to adopt the stringent criteria for detecting favored and hitchhiking mutations. Fourth, since fewer GWAS studies have been conducted with African populations, associated favored mutations and GWAS sites are underestimated in African populations. Future versions will report more favored and hitchhiking mutations and updated data from related sources.

## CRediT authorship contribution statement

J.T. and Hao Zhu designed the study. J.T., Huanlin Zhang, and Hai Zhang built the database and the website. J.T and Hao Zhu wrote the manuscript.

## Declaration of Competing Interest

The authors declare no competing interests.
